# Non-contact radiofrequency-induced reduction of subcutaneous abdominal fat correlates with initial cardiovascular autonomic balance and fat tissue hormones: safety analysis

**DOI:** 10.12688/f1000research.5708.1

**Published:** 2015-02-20

**Authors:** Jiri Pumprla, Kinga Howorka, Zuzana Kolackova, Eliska Sovova

**Affiliations:** 1 International Research Group Functional Rehabilitation & Group Education, Vienna, 1090, Austria; 2 Vila Krasy Aesthetic Centre, Internal Medicine Outpatient Clinic, Olomouc, 779 00, Czech Republic; 3 Center of Medical Physics and Biomedical Engineering, Medical University Vienna, Vienna, 1090, Austria; 4 Internal Medicine/Diabetology Clinic, Prevention and Aesthetics Centre, Vienna, 1170, Austria; 5 Department of Internal Medicine I and Department of Sports Medicine and Cardiovascular Rehabilitation, University Palacky Medical School, Olomouc, 771 47, Czech Republic

**Keywords:** Metabolic syndrome, insulin resistance, selective-field radiofrequency, body contouring, subcutaneous fat reduction, heart rate variability, autonomic control

## Abstract

**Background and objective: **The non-invasive reduction of subcutaneous abdominal fat became popular in the last decade. Radiofrequency (RF), non-contact, selective-field device Vanquish® has been developed to selectively induce deep fat tissue heating to reduce waist circumference. Our analysis evaluates immediate and sustained effects of this treatment on cardiovascular autonomic function and on selected metabolic parameters.

**Study design/** **patients and methods: **A retrospective proof-of-concept analysis of RF treatment effects was conducted in 20 individuals with metabolic syndrome, to reduce the subcutaneous abdominal fat. Four 30-minutes treatment sessions (manufacturer´s standard protocol) were performed in 1-week intervals. Vital signs, ECG, lab screening, body composition, subcutaneous fat thickness and spectral analysis of heart rate variability (HRV) have been examined before, after the 1 ^st^ and 4 ^th^ treatment, and at follow-up visits 1 month and 3 months after the treatment.

**Results:** The RF treatment led to a significant reduction of abdominal circumference after the 4 ^th^ session (p<0.001), and during follow-up after 1 and 3 months (p<0.001 and p<0.02, resp.). There was a significant correlation (r=-0.58, p=0.007) between reduction of abdominal circumference and initial very-low frequency (VLF) spectral power at 1 month follow-up. A significant increase of cumulative spectral power in low frequency (p=0.02) and reduction in high frequency (p=0.05) band have been observed immediately (20 +14 minutes) after the treatment. On the contrary, no sustained impact on autonomic balance has been recorded 39 +18 days after the treatment. A significant correlation between the initial adiponectin values and immediate autonomic response to one treatment was observed in VLF and total spectral bands (r>0.59, p<0.04).

**Conclusions:** Our analysis shows that the selective-field RF treatment is safe and efficient for reduction of subcutaneous abdominal fat. While the treatment increases the immediate sympathetic response of the body to deep tissue heating, no sustained change in autonomic function could be recorded at 1 month follow-up. The observed correlation between initial VLF spectral power and waist circumference reduction at follow-up, as well as the association of initial adiponectin values and immediate autonomic response to the treatment might be instrumental for decisions on body contouring strategies.

## Introduction

Obesity considerably impairs individual health and aesthetic appearance. In addition to its known health impacts -- reduction of life expectancy and quality of life ^1^ -- obesity leads to numerous problems including disadvantages in employment ^2^, in social interactions and decreased satisfaction with own body image ^3^. These aspects lead to social pressure and subsequently to increased demand for effective procedures for weight reduction, body contouring and beauty enhancement.

Central obesity is associated with insulin resistance and related components of metabolic syndrome that can be typically treated by nutritional, behavioural and lifestyle changes ^1^. Although the reduction of subcutaneous fat alone does not lead directly to reduction of cardiovascular risk in obese subjects ^4^, there is some evidence that the large-volume liposuction might positively influence the insulinemia ^5^ and thus insulin sensitivity. Furthermore, clinical experience shows that aesthetic procedures leading to improved patient’s self esteem ^6^ often significantly enhance the motivation to further lifestyle changes towards healthier goals.

Various invasive and particularly non-invasive body contouring procedures for reduction of subcutaneous fat layers have been introduced in the last decade. While the surgical liposuction still counts for the most effective gold-standard procedure in this respect ^7^, due to its invasiveness, downtime and side effects, a bunch of non- or semi-invasive procedures became available as its indirect alternative on the quickly growing (often called “lunch-time-procedure”) market ^8^. However, despite many individual – often only anecdotal – user reports, only a minority of methods is proven according to the evidence-based medicine standards. Such evidence is available for efficacy of chemical lipolysis, based on injection of phosphatidylcholine and deoxycholic acid ^9^, and for selected energy-based technologies using focused ultrasound ^10^, cryolipolysis ^11^ and/or thermal/radiofrequency for lipolysis ^12^. Despite the broad use in the practice, clinical safety data of these aesthetic procedures are scarce, with only a few publications available ^13^. Although these intensive procedures might have a significant impact on autonomic homeostasis and individual health, to our knowledge, no immediate or sustained effects of such treatments on autonomic function have been investigated.

Analysis of beat-to-beat fluctuations of heart rate (heart rate variability, HRV) is an established tool to non-invasively quantify cardiac autonomic function ^14^. The frequency (spectral) decomposition and quantification of irregular course of heart rate into three main frequency bands allows a detailed view of different domains of the cardiovascular control. The short-term HRV spectral analysis is proven useful for assessment of impact of various physiological stimuli on the body such as food restriction ^15^, endurance physical training ^16^ or guided breathing ^17^. In particular, the very low frequency (VLF) spectral band has been shown to reflect thermoregulatory vasomotor mechanisms, changes in peripheral chemoreceptor activity and fluctuations in renin-angiotensin systems ^14, 18^. In this respect, analysis of the VLF band enables the quantification of sympatho-thermogenic autonomic responses related to energy metabolic control, as it has been demonstrated e.g. by an acute cold exposure, spicy food containing capsaicin and green tea extract or low-calorie diet ^19– 22^.

The selective-field radiofrequency device Vanquish ^®^, using electromagnetically induced rapid oscillations of electrical dipoles to heat up the fatty tissue ^23^, is increasingly being used for reduction of subcutaneous abdominal fat. Its efficacy has already been demonstrated ^24^. However, although the reported patient acceptance of these treatments was well to superlative ^24^, no metabolic and/or safety data have been published yet. Our aim was therefore to evaluate the safety and efficacy of this novel technology in a proof-of-concept retrospective data analysis of all clients who attended our clinic and were subjected to the treatment including follow-up within the 5-months time period. This paper focuses on the immediate and sustained effects of the treatment on the autonomic balance of the body and related metabolic values. The analysis is thought as a preparation for a further controlled prospective observation.

## Patients and methods

### Study design

A retrospective, uncontrolled, single site proof-of-concept analysis of the impact of selective-field radiofrequency treatment on cardiovascular autonomic control and on selected metabolic data (insulin resistance parameters and fat tissue hormones) was conducted in overweight individuals with components of metabolic syndrome and visually detectable excess of subcutaneous fat who wished to reduce the abdominal circumference. This study was a retrospective proof-of-concept trial and the data have been retrospectively evaluated for the future preparation of a controlled prospective trial, which will require a submission to the local Ethics Committee. For the retrospective data evaluation no approval of IRB was required.

Data have been routinely acquired before, at visits immediately after the 1 ^st^ and 4 ^th^ treatments, and at follow-up visits in 1-month and 3-months after the last treatment. For assessment of metabolic data, blood sampling was performed before, on the next morning after the 1 ^st^ and 4 ^th^ treatments, and 1 and 3 months after the last treatment. Assessment of the intervention effect on the autonomic balance, using the standardized analysis of short-term heart rate variability as obtained during the modified orthostatic load ^18, 25^, was performed before, immediately (acute effect) after the 1 ^st^ treatment, and 1 month (sustained effect) after the last treatment. These data have been acquired during routine services of the clinic.

### Inclusion and exclusion criteria

The selective-field RF treatment protocol has been offered to all individuals with visually excessive subcutaneous fat wishing to reduce their waist circumference. In the retrospective evaluation of efficacy and safety all patients have been included who accepted the necessity of follow-up investigations. Attendance of follow-up visits was a prerequisite for waiving their treatment fees. No reimbursement or coverage of travel expenses have been offered to these patients.

The following standard routine criteria of our clinic for exposition to RF treatment were applied: *Inclusion* criteria were age 20–70 years, both genders, BMI over 25kg/m ^2^, abdominal circumference over 80 and 94 cm in women and men, respectively, with at least 20 mm of abdominal subcutaneous adipose tissue (as measured by calliper at predefined locations), stable weight over the last 6 months and signed informed consent on treatment. *Exclusion* criteria were pregnancy or insufficient contraceptive methods, surgical liposuction within the last 12 months, insufficiently controlled metabolic disease including diabetes mellitus of both types, untreated hypo- or hyperthyroidism, uncontrolled liver, kidney or cardiovascular disease, implanted pacemaker or metal implant, acute or feverish disease, history of thrombophlebitis, any haematological disease, chronic medication of corticosteroids, beta-blockers, anticoagulants, insufficient treatment adherence or any other clinical or biochemical condition bearing potential to interfere with the treatment targets. Females in child-bearing age were educated about necessary contraceptive methods, and those planning pregnancy in the following 12 months were not subjected to the RF treatment.

### Patients

The study population consisted of n=20 (f=18/m=2) subjects with age 47.8±7.2yr, BMI 28.2±3.6 kg/m ^2^, abdominal circumference 96±9 cm, insulin resistance HOMA2 index 1.49±0.80 with insulin sensitivity of 79.8±28.9%, fat percentage in body composition 38±7%, blood pressure 138±12/79±7 mmHg, and with reported insufficient aerobic activity/median 30/Q1=0, Q3=60/min weekly. Chronic treatment of concomitant diseases remained unchanged during the whole treatment period. Six female patients received substitution of hypothyroidism resulting in euthyroid values of TSH (x=1.2±0.8 mU/l), four subjects used antihypertensive medication (ACE inhibitors or sartans) and four subjects had lipid lowering agents (statins). Eight female patients received oral contraceptives. Further details can be found in Table 1.

**Table 1.** Clinical characteristics of the study population.

	mean±SD
**Abdominal circumference (cm)**	
minimum	84.6±9.2
umbilicus	96.2±9.3
5cm below umbilicus	101.2±9.9
**Calliper/Skinfold thickness (mm)**	
spina illiaca left (mm)	18±4
spina illiaca right	19±4
1/3 between SI and umbilicus left	24±4
1/3 between SI and umbilicus right	25±4
5cm below umbilicus	23±5
**Body composition**	
weight (kg)	78.8±12.4
height (cm)	166.9±7.2
BMI (kg/m ^2^)	28.2±3.6
body fat (%)	38.1±7.2
muscle (%)	27.1±3.6
basal metabolic rate (kcal)	1535.7±178.5
visceral fat (arbitrary units)	8.6±2.4

### Intervention

The non-contact, selective-field radiofrequency system Vanquish ^®^ (BTL Industries) has been used for treatment of subcutaneous fat layers ^23^. All subjects underwent four 30 minutes treatment sessions in the abdominal area, with one week break between the sessions, as recommended in the standard treatment protocol by the manufacturer. Flat multipolar applicator panel was used for emitting the radiofrequency (27.12 MHz) energy for selective generation of deep tissue thermal heating of adipose tissue layers. The unit adjusts the parameters of the emitted energy in real time and shows the instantaneous value on display. This electromagnetic radiation is heating up the adipose tissue much more effectively then surrounding tissues, while limiting potential side effects due to minimized exposition of skin, muscles, or internal organs to this energy ^23^. The treatment procedure consists of placing the emitting panel over abdomen and flanks close to the skin using a spacer which standardizes distance between the panel and the body surface. Once it is in a proper position, treatment can be started while the intensity of the emitted energy is set according to the protocol and to tuning efficiency of the system. The skin temperature is measured before, in 10, 20 and 30 minutes during the treatment, while the subject is frequently asked to give feedback on subjective thermal perception and to immediately report any pain or unpleasant sensations. Operator adjusts the emitted energy intensity close to the tolerable level according to client’s feedback during the treatment procedure and to the measured skin temperature while the safety threshold is set to 42°C.

### Routine clinical assessments

Blood sampling, autonomic balance evaluated by heart rate variability analysis, clinical assessment including vital signs, casual blood pressure, electrocardiogram (ECG), body composition evaluated by bioelectrical impedance measurements, abdominal circumference in three predefined points and anthropometric assessment by calliper were evaluated at predefined time points as indicated elsewhere.

### Measurement of vital signs

Casual blood pressure has been measured in accordance with standard recommendations ^26^ in sitting position, using validated oscillometric automated monitor Omron M6 (Omron, Japan). The average from three measurements has been used for data analysis.

A calibrated, computer-assisted system ECG Seiva (Seiva, Czech Republic) has been used for 12-lead surface ECG recordings. Data have been electronically stored and evaluated by a single specialist experienced in ECG readings.

Height has been measured by validated ultrasound height measuring unit ADE MZ10020 (ADE, Germany) within standardized conditions as set by manufacturer.

Temperature before and during the treatment sessions has been measured by a calibrated non-contact infrared skin thermometer BaseTech IRT-350 (BaseTech, Germany) at predefined locations (around umbilicus and at upper and lower abdominal wall on both sides).

Weight has been measured within the body composition assessment as described below.

### Assessment of weight and body composition

Body composition has been assessed by a calibrated scale, Omron BF 511 (Omron, Japan), a 8-sensor, one-frequency (50 kHz, 500 uA) bioelectrical body impedance analysis device, under strictly standardized conditions as set by the manufacturer. The device delivers along with weight and BMI also gender-specific percentage of body fat and muscle mass, basal metabolic rate (in kcal) and amount of visceral fat (arbitrary units). The declared weight measurement accuracy is 1% ^27^.

### Assessment of waist circumference

Waist circumference was measured using a measuring tape with a spring handle ( www.netzwerk-lipolyse.de), in order to control for the pressure exerted on the patient’s abdomen. Three measurements in different locations have been performed at the end of gentle expiration, in the standing position: horizontally around the patient’s abdomen at its narrowest part (under the rib cage), at the level of the umbilicus, and 5 cm below the umbilicus. Data were recorded to the nearest millimetre.

### Assessment of subcutaneous fat layer using calliper (skinfold thickness measurements)

All measurements were performed with the subject in standing position. The measurement points were selected as follows: above the iliac crest in the mid-axillary line, right and left, paraumbilically at 1/3 distance between the iliac crest and umbilicus, right and left, and 5 cm below umbilicus. The skinfold was pinched up firmly between the thumb and forefinger and pulled away from the underlying tissues. The measurements were performed with calibrated calliper of Harpenden type, ie. with a constant measuring pressure 10p/mm ^2^, in accordance with established guidelines ^28^. The results are presented in mm, as average of five subsequent measurements per one point.

A standard blood sampling has been performed in the morning by venipuncture after an overnight 10 hours fasting. After clotting, the serum was separated and immediately explored for most analyses. For fat hormones, the serum was stored at -20°C until analysed. Insulin resistance was evaluated using the HOMA2 calculations based on fasting glycemia and C-peptide values (Homeostatic model assessment as described by Levy *et al.* ^29^).

### Assessment of autonomic balance

A standardized analysis protocol of short-term HRV in time and frequency domain as obtained during a modified orthostatic load (5 minutes supine and 5 minutes in standing position) has been used for quantification of treatment effects on the autonomic control of the body ^14, 18, 25^. The HRV measurements have been performed using the VariaCardio TF5 system (Advanced Medical Diagnostics Group, UK). The main principle of spectral analysis of HRV is a decomposition (using fast Fourier transform algorithms) of irregular fluctuations of heart rate into regular cycles that represent influences of various domains on the autonomic balance. Such resulting spectral power is then quantified within three standard frequency bands: (1) very-low frequency component (VLF, 0.01–0.04 Hz), its cycles occur with typical frequency of 0.01 Hz, corresponding to wavelength of 100 seconds. The VLF power is related to control of energy metabolism and thermoregulation, changes in peripheral chemoreceptor activity and fluctuations in renin-angiotensin system, (2) low-frequency component (LF, 0.04–0.15 Hz), with typical variations occurring at frequency 0.1 Hz, i.e. 6-times per minute. It represents predominantly sympathetic control with certain amount of vagal influence, (3) high-frequency component (HF, 0.15–0.4 Hz), with cycles fluctuating at average frequency 0.25 Hz, ie 15-times per minute. This power is related to respiratory activity and parasympathetic control ^14^. While the VLF band is mediated primarily by sympathetic control and the HF by the parasympathetic one, the middle one, LF band, includes both, with predominance of the sympathetic branch of the autonomic control ^14, 18, 30^. The main parameters of the analysis are the spectral power (area under the curve) in each of the individual bands and in the total frequency band, the centroid frequencies and the relative proportion of individual frequency bands contents in the total spectral power. We have shown previously that the cumulative numbers generated by summing up the individual frequency band spectral powers over both test positions increase the discrimination power/capability of respective parameters ^32^.

### Statistical analysis

Statistical analysis was performed using standard statistical packages (SPSS, Statistical Package for the Social Sciences V10.0, SPSS Inc., Chicago, USA). Normality of data distribution was verified by Kolmogoroff-Smirnoff test. A two-tailed paired Student’s t-test was applied to estimate differences between groups in case of normal data distribution. Relations among variables were assessed using Pearson’s correlation analysis. Data are presented as means ± SD, unless indicated otherwise. The significance level was set a priori at p<0.05.

## Results

### Treatment intensity

During all four sessions, the average skin temperature values before, at 10, 20 and 30 minutes of treatment were 31.8±1.1, 39.8±0.7, 39.6±0.6 and 39.2±1.0°C respectively, while the delivered total average maximum energy was 158.5±13.0 W and the total average effective energy was 156.2±13.1 W. While starting the treatment session at 160 W energy level as suggested by manufacturer, in 25 out of 84 (29,8%) sessions the energy intensity could be increased -- in accordance with subject’s heat sensation -- to 170–200 W within the first 10 minutes of treatment, and in 13 out of 84 (15.5%) sessions the energy intensity had to be reduced to 100–150 W due to excess heat perception. The average effective emitted energy in each of four treatment session was therefore 156±14, 160±17, 160±19, and 153±16 W respectively. A significant correlation between the averaged skin temperature after 30 minutes of treatment and reduction in abdominal circumference was observed 1 month after the last treatment (r=-0.49, p=0.03).

### Vital signs

When compared with initial values, the average casual blood pressure was significantly lower after the 4 ^th^ treatment session (134±12 vs. 127±10 mmHg, p=0.003) and raised to 129±9 mmHg (p=0.04 vs. initial value) 1 month after the treatment. The average heart rate has changed from 69±12 to 67±9/min (p=0.04) after 4 ^th^ treatment session, and raised to 69±11/min (p=0.33, both p vs. initial value) after 1 month. No other significant changes have been observed in ECG.

### General effects

The radiofrequency selective-field treatment lead to a significant reduction of abdominal circumference as measured at 3 different locations after the 4 ^th^ session (umbilicus, 96.2±9.3 vs 93.7±9.0 cm, p<0.001 vs. initial value), and during follow-up after 1 month (92.6±9.6 cm, p<0.001) as well after 3 months (93.3±10.1 cm, p<0.02). Despite the significant drop in body weight at follow-up 1 month after the treatment (from 78.8±12.4 to 78.0±12.1 kg, p=0.001), no significant correlation has been found between the deltas in body weight and abdominal circumference values vs. their respective initial values at this time point (r<0.41, p>0.07). The weight increased to 78.4±12.0 kg after 3 months follow-up. No statistically significant change in body composition (in percentage of body fat and muscle mass) has been recorded during all three measurements vs. initial values.

### Autonomic balance

**Figure 1.**
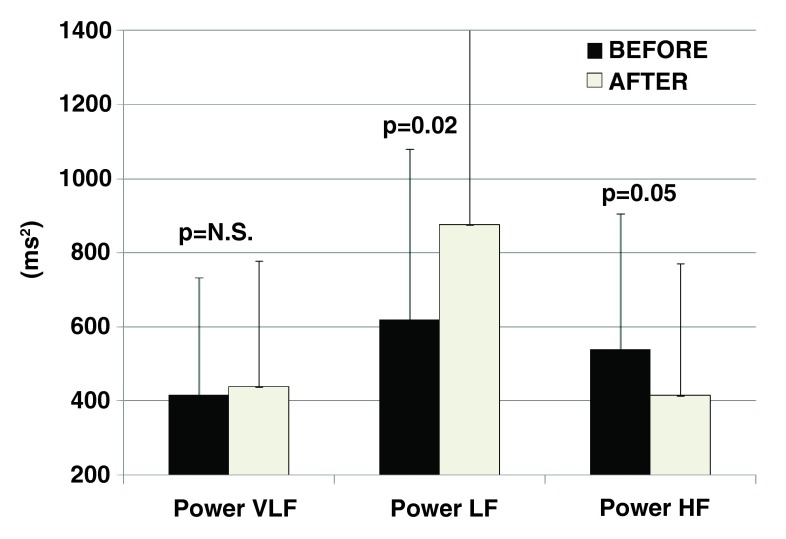
Immediate effects of a single session with Vanquish ^®^ RF treatment in abdominal area on heart rate variability (cumulative spectral power in very low/Power VLF/, low/Power LF/ or high/Power HF/frequency bands): significant increase of predominantly sympathetic **(LF)** and decrease in parasympathetic **(HF)** components 20±14min after the treatment.

**Figure 2.**
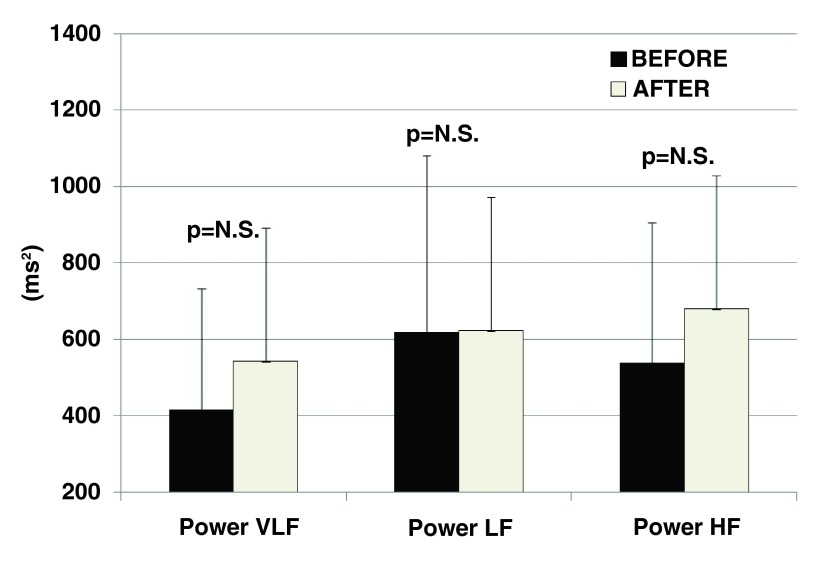
No sustained effects of a series of 4 treatments with Vanquish ^®^ RF treatment on heart rate variability as assessed 39±18 days after the last treatment session. For description of spectral parameters see Figure 1.

Regarding the immediate effects of the treatment on autonomic balance, a significant increase in low frequency (p=0.02) and reduction in high frequency (p=0.05) band cumulative spectral powers have been observed in HRV 20±14 minutes after the treatment. No sustained effects on autonomic balance, however, have been observed during the follow-up period after the treatment. Figure 1 and Figure 2 summarize the impact of the treatment on autonomic balance immediately after one treatment and 39±18 days (sustained effect) after the last treatment, respectively.

**Figure 3.**
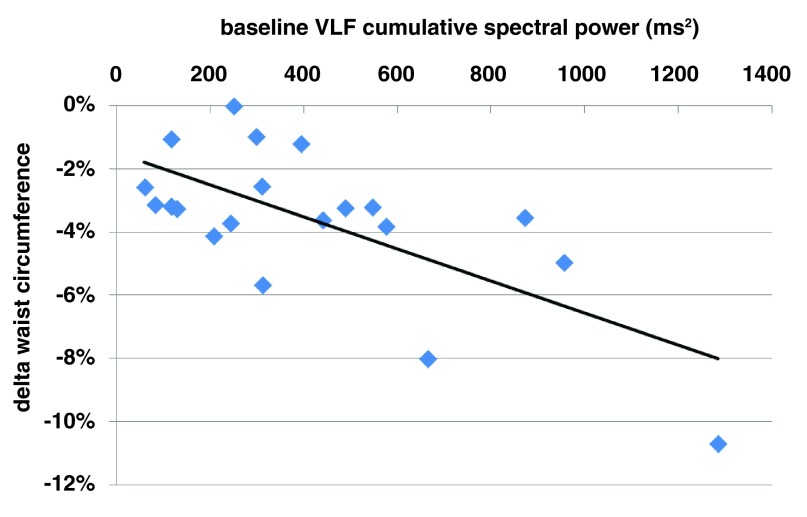
Treatment efficacy as assessed by delta waist circumference 1 month after the last treatment significantly correlates with initial VLF (very-low frequency) cumulative spectral power (r=-0.58, p<0.001).

At follow-up after 1 month, there was a significant correlation between the reduction of abdominal circumference and the initial very-low frequency band cumulative spectral power (r=-0.58, p=0.007, Figure 3). Moreover, in a subgroup comparison, subjects with a higher initial cumulative VLF power (6.4±0.4 LN ms ^2^) demonstrated a significantly bigger drop in abdominal circumference after the 4 ^th^ treatment (4.1±1.9 vs. 2.6±0.9 cm, p=0.045) than those with lower initial cumulative VLF spectral power (5,1±0.5 LN ms ^2^).

### Interrelationships between treatment effects and metabolic parameters

As expected, a significant correlation between weight and insulin resistance index based on HOMA2 calculations has been observed before the treatment (p=-0.53, p=0.016). Change of body weight correlated significantly with the initial HOMA2 indices after the 4 ^th^ treatment (r=-0.54, p=0.014 for HOMA2, and r=-0.47, p=0.036 for % beta function) and 1 month after the last treatment (p=-0.57, p=0.009 for % beta function). There was a significant correlation between the initial adiponectin values and deltas of total body fat percentage (r=-0.45, p=0.05) and body weight (r=-0.52, p=0.02) observed after the 4 ^th^ treatment.

### Metabolic and autonomic interrelationships

**Figure 4.**
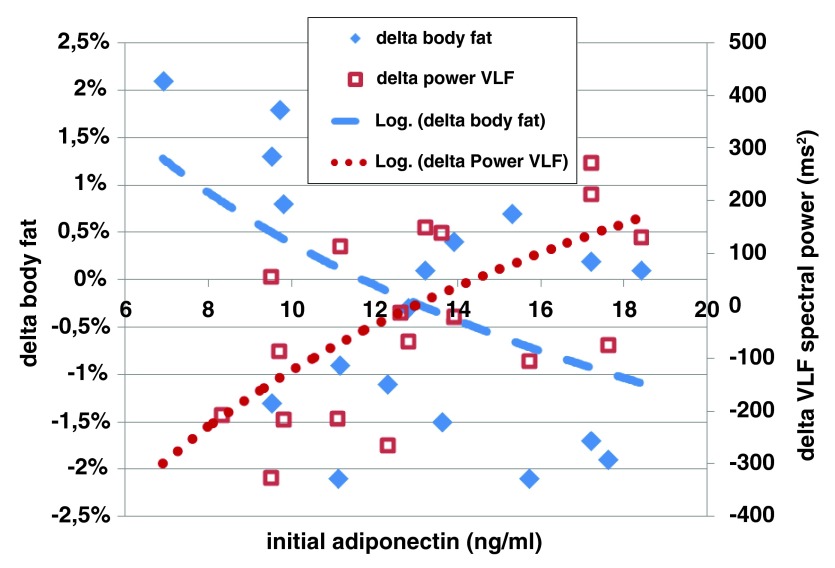
Significant correlations between baseline adiponectin values and immediate autonomic response at VLF band to single treatment (r=0.60, p=0.005) and between baseline adiponectin values and change of body fat percentage after series of 4 treatment sessions (r=-0.45, p=0.05).

The immediate autonomic response to one treatment ( Figure 4), as observed in the VLF band (r=0.60, p=0.005) and in the total spectral power (r=0.45, p=0.04) correlated significantly with the initial adiponectin values. Furthermore, a subgroup analysis based on initial adiponectin values (cut-off value 13.0 ng/ml) revealed a significantly stronger acute autonomic response to one treatment in those with higher initial adiponectin level (15.8±1.8 ng/ml) than with a lower one (10.3±1.8 ng/ml). Similarly, in respect to sustained effects, there was a significant correlation between delta of adiponectin values 1 month follow-up vs. initial values and delta of autonomic response in VLF band 1 month follow-up vs. initial values (r=0.48, p=0.03).

### Side effects and drop-outs

Overall, two drop-outs have been recorded. In two cases the local skin irritation led to interruption of the protocol after the 2 ^nd^ treatment. These subjects were not included in the analysis. One subject underwent elective surgery at 1 month of follow-up and did not attend the planned visit. Four subjects did not attend the last follow-up visit after 3 months.

After first two treatments, one subject reported abdominal discomfort, and another one a hyperesthesia around umbilicus. These symptoms resolved within 1 week after the treatment session. No more adverse reactions have been observed after exchange of spacer used for proper positioning of the energy emitting panel at the 3 ^rd^ treatment session.

Data of non-contact radiofrequency-induced reduction of abdominal fat HRV The heart rate variability data are provided.

## Discussion

The principal findings of our study are threefold: the efficacy of selective-field radiofrequency treatment in terms of reduction of waist circumference up to 3 months after the treatment series was confirmed. The treatment is safe, as no clinically relevant side-effects were observed. The impact on autonomic cardiovascular balance is significant but transient, while being limited to an increased sympathetic response immediately after this energy-based treatment in abdominal area. No sustained effect of the intervention on autonomic balance has been observed 1 month after the last treatment. The treatment efficacy is inversely associated with insulin resistance and other features of metabolic syndrome and may be explained by the inhibitory effect of higher insulin levels on the (treatment-induced) lipolysis ^32^. Whether the treatment efficacy could be better predictable using assessment of VLF spectral power and insulin resistance profile, and/or it could be further supported e.g. by pharmacological agents (such as metformin or insulin sensitisers ^33^) or other means, this should be investigated in further prospective trials.

There is a pathogenic link among autonomic imbalance, insulin resistance and obesity. In addition to genetic background and lifestyle factors, autonomic imbalance could be a common root of obesity, hypertension and/or type 2 diabetes mellitus. At early stages of obesity/metabolic syndrome development, parasympathetic control is decreased while sympathetic overactivity usually occurs ^34^. This dysfunction increases cardiovascular workload, hemodynamic stress and induces potentially significant cardiac pathology leading to serious arrhythmias. It remains open, however, whether elevated sympathetic tone is a *primary* feature that contributes to the development of obesity and metabolic syndrome or if it develops and/or changes *secondary* to the obese state.

It has been shown that sympathetic overactivity precedes the development of insulin resistance and type 2 diabetes mellitus ^35^. Studies with genetically predisposed humans with insulin resistance have shown that early insulin resistance is already associated with increased sympathetic control, and it has been suggested that hyperinsulinemia is the initiating factor leading to increase of sympathetic neural activity ^36^. Subsequently, adrenoceptor down-regulation and/or reduced sensitivity are likely to develop which situation results in a secondary reduction of sympathetic responsiveness. As adrenoceptors are involved in control of energy expenditure, their down-regulation leads further to impaired food-induced thermogenesis and post-prandial fat oxidation, promoting the accumulation of body fat. In this way, the development of obesity can be seen as a consequence of inappropriate/insufficient sympathetic control, energy dissipation, gaining weight and then insulin resistance ^37^. This theory also confirms the earlier popular Bray’s MONALISA hypothesis, stating that *“Most Obesities kNown Are Low In Sympathetic Activity”* ^38^. It is also consistent with findings from population studies, e.g. in observation of 7000 individuals without hypertension at baseline, low heart rate variability predicted greater risk of incident hypertension over 9 years of follow-up ^39^. Similarly, in almost 2000 participants of Framingham Offspring Study, LF power and LF/HF ratio were lower in diabetic subjects than in those with normal fasting glucose. HRV was inversely associated with plasma glucose levels and was reduced in diabetic individuals as well as in subjects with impaired fasting glucose levels ^40^.

Heart rate variability measurement is an established tool for the assessment of impact of intervention on autonomic balance ^15– 17, 41, 42^. While the HRV LF and HF frequency bands have been sufficiently studied in short- and long-term ECG recordings, interpretation of the VLF band -- particularly in short-term recordings – is less explored. Along with influences coming from sympatho-thermoregulation, renin-angiotensin system and chemoreceptors, a clear VLF response to excessive temperature variations has been demonstrated ^19^. Further on, significant impacts of a spicy food ^20^ or capsaicin ^21^ on VLF spectral power have been reported. These findings are consistent with our results where a significant correlation has been observed between the initial adiponectin level and the immediate VLF band autonomic response to a single treatment, as well as between the initial adiponectin and reduction of percentage of body fat after the treatment series ( Figure 4). Additionally to fat percentage, the initial VLF spectral power significantly correlated with change in waist circumference seen after the treatment series ( Figure 3). These observations raise a question whether individuals with higher VLF spectral power and higher adiponectinemia/lower insulin resistance might enjoy a better sympatho-thermogenic capability to “burn” the available energy while more readily inducing lipolysis processes, than those individuals with lower VLF tone. This hypothesis might have clinical implications in weight management programs and/or body contouring treatments for subcutaneous fat layers reduction.

At present, there are some new therapeutic targets and procedures taking into account autonomic imbalance in obesity as an independent and sensitive marker of health. Autonomic dysfunction is reversible with lifestyle changes such as hypocaloric nutrition or fasting ^15^ and physical endurance training ^16^. Recently, the approach of cold-induced, facultative thermogenesis aiming for sympathoadrenergically-mediated weight reduction by stimulation of brown fat tissue has been introduced ^43, 44^. A sympathetic stimulation of brown fat tissue leading to increased daily energy expenditure by 200–400 kcal ^45^ has been suggested as the main mechanism in this successful model, and there is some hope for success of this approach on the reduction of obesity ^46^. The conversion of white adipose tissue to the highly thermogenic beige adipose tissue has been shown to be influenced by acute sympathetic activation, as well ^47^. Since the autonomic imbalance is a marker of adverse risk ^14, 18^, its improvement resulting from weight loss should be beneficial for the health of obese/diabetic individuals.

Our relatively short and limited observation could not deliver sufficient evidence on whether subcutaneous abdominal fat reduction using selective-field RF treatment improves obesity-related cardiovascular risk. However, despite even only little changes in body weight, patients with significantly reduced waist circumference are reported to have an improved metabolic profile ^48^. It has been shown that the waist circumference is a proven marker of higher total mortality risk ^49^ as well as of a cardiovascular risk ^50^. Therefore, reducing waist circumference and percentage of fat in body composition may represent a useful and clinically relevant target ^48^. Moreover, the initial successful waist reduction may play a significant role in further stimulating the motivation of patients with metabolic syndrome for long-term adaptation and adherence to “healthier” lifestyle habits. As this is a proof-of-concept uncontrolled retrospective analysis, we cannot fully exclude external confounding factors that might have contributed to our observations. As a logical step, a randomized, controlled trial verifying the results in an appropriate patient sample would contribute to a better understanding of our findings.

In conclusion, our analysis provided a proof of concept for safety and efficacy of selective field RF treatment using the standard 4 × 30 minutes protocol for moderate reduction of subcutaneous fat tissue. Only transient and non-sustained effects on autonomic balance have been found during the follow-up after the treatment series. The efficacy of Vanquish RF treatment in terms of waist circumference reduction was shown and it was significantly related to initial VLF spectral power and adiponectin levels. This implicates that less insulin resistance may offer better conditions for lipolytic action of the treatment. This body contouring procedure was the most efficient in moderate abdominal overweight with lower insulin resistance, and as such can well complement other, systemic clinical measures for weight reduction based on lifestyle and nutritional changes. As the measurements of HRV and fat hormone status are easily performed, we suggest considering the inclusion of these parameters into the clinical prescreening armamentarium in order to enhance the outcomes of aesthetic body contouring methods.

## Consent

Written informed consent for publication of their anonymised clinical details was obtained from all patients.

## Data availability

F1000Research: Dataset 1. Data of non-contact radiofrequency-induced reduction of abdominal fat HRV, 10.5256/f1000research.5708.d38309 ^51^

## References

[1] European Guidelines on cardiovascular disease prevention in clinical practice (version 2012): The Fifth Joint Task Force of the European Society of Cardiology and Other Societies on Cardiovascular Disease Prevention in Clinical Practice (constituted by representatives of nine societies and by invited experts). *Atherosclerosis.* 2012;223(1):1–68. 10.1016/j.atherosclerosis.2012.05.007 22698795

[2] Puhl RM Heuer CA: The stigma of obesity: a review and update. *Obesity (Silver Spring).* 2009;17(5):941–964. 10.1038/oby.2008.636 19165161

[3] Jaworowska A Bazylak G: An outbreak of body weight dissatisfaction associated with self-perceived BMI and dieting among female pharmacy students. *Biomed Pharmacother.* 2009;63(9):679–92. 10.1016/j.biopha.2008.08.005 19179040

[4] Klein S Fontana L Young VL: Absence of an effect of liposuction on insulin action and risk factors for coronary heart disease. *N Engl J Med.* 2004;350(25):2549–2557. 10.1056/NEJMoa033179 15201411

[5] Boriani F Villani R Morselli PG: Metabolic effects of large-volume liposuction for obese healthy women: a meta-analysis of fasting insulin levels. *Aesthetic Plast Surg.* 2014;38(5):1050–6. 10.1007/s00266-014-0386-3 25099498

[6] Rubesa G Tic-Bacić T Svesko-Visentin H: The influence of aesthetic surgery on the profile of emotion. *Coll Antropol.* 2011;35(Suppl 2):51–5. 10.1016/j.eurpsy.2007.01.577 22220403

[7] Matarasso A Levine SM: Evidence-based medicine: liposuction. *Plast Reconstr Surg.* 2013;132(6):1697–705. 10.1097/PRS.0b013e3182a807cf 24281595

[8] Katz BE Sadick NS: Body contouring. Procedures in Cosmetic Dermatology. Saunders Elsevier,2010;202 Reference Source

[9] Reeds DN Mohammed BS Klein S: Metabolic and structural effects of phosphatidylcholine and deoxycholate injections on subcutaneous fat: a randomized, controlled trial. *Aesthet Surg J.* 2013;33(3):400–8. 10.1177/1090820X13478630 23439063 PMC3667691

[10] Shalom A Wiser I Brawer S: Safety and tolerability of a focused ultrasound device for treatment of adipose tissue in subjects undergoing abdominoplasty: a placebo-control pilot study. *Dermatol Surg.* 2013;39(5):744–51. 10.1111/dsu.12123 23432811

[11] Krueger N Mai SV Luebberding S: Cryolipolysis for noninvasive body contouring: clinical efficacy and patient satisfaction. *Clin Cosmet Investig Dermatol.* 2014;7:201–5. 10.2147/CCID.S44371 25061326 PMC4079633

[12] Anolik R Chapas AM Brightman LA: Radiofrequency devices for body shaping: a review and study of 12 patients. *Semin Cutan Med Surg.* 2009;28(4):236–43. 10.1016/j.sder.2009.11.003 20123422

[13] Klein BK Zelickson B Riopelle JG: Non-invasive cryolipolysis for subcutaneous fat reduction does not affect serum lipid levels or liver function tests. *Lasers Surg Med.* 2009;41(10):785–790. 10.1002/lsm.20850 20014252

[14] Heart rate variability: standards of measurement, physiological interpretation and clinical use. Task Force of the European Society of Cardiology and the North American Society of Pacing and Electrophysiology. *Circulation.* 1996;93(6):1043–1065. 10.1161/01.CIR.93.5.1043 8598068

[15] Howorka K Pumprla J Schabmann A: Influence of fasting on heart rate variability in diabetic patients with different degrees of cardiovascular autonomic neuropathy. *Diab Nutr Metab/Clin Exp.* 1997;10:288–295.

[16] Howorka K Pumprla J Haber P: Effects of physical training on heart rate variability in diabetic patients with various degrees of cardiovascular autonomic neuropathy. *Cardiovasc Res.* 1997;34(1):206–214. 10.1016/S0008-6363(97)00040-0 9217892

[17] Howorka K Pumprla J Tamm J: Effects of guided breathing on blood pressure and heart rate variability in hypertensive diabetic patients. *Auton Neurosci.* 2013;179(1–2):131–7. 10.1016/j.autneu.2013.08.065 24021938

[18] Pumprla J Howorka K Groves D: Functional assessment of heart rate variability: physiological basis and practical applications. *Int J Cardiol.* 2002;84(1):1–14. 10.1016/S0167-5273(02)00057-8 12104056

[19] Friedman BH Thayer JF Tyrrell RA: Spectral characteristics of heart period variability during cold face stress and shock avoidance in normal subjects. *Clin Auton Res.* 1996;6(3):147–52. 10.1007/BF02281901 8832123

[20] Matsumoto T Miyawaki C Ue H: Comparison of thermogenic sympathetic response to food intake between obese and non-obese young women. *Obes Res.* 2001;9(2):78–85. 10.1038/oby.2001.10 11316350

[21] Shin KO Moritani T: The combined effects of capsaicin, green tea extract and chicken essence tablets on human autonomic nervous system activity. *J Nutr Sci Vitaminol (Tokyo).* 2007;53(2):145–52. 10.3177/jnsv.53.145 17616002

[22] Fujibayashi M Hamada T Matsumoto T: Thermoregulatory sympathetic nervous system activity and diet-induced waist-circumference reduction in obese Japanese women. *Am J Hum Biol.* 2009;21(6):828–35. 10.1002/ajhb.20899 19384859

[23] Weiss R Weiss M Beasley K: Operator independent focused high frequency ISM band for fat reduction: porcine model. *Lasers Surg Med.* 2013;45(4):235–9. 10.1002/lsm.22134 23619902 PMC3664404

[24] Fajkosova K Machovcova A Onder M: Selective radiofrequency therapy as a non-invasive approach for contactless body contouring and circumferential reduction. *J Drugs Dermatol.* 2014;13(3):291–296. 24595574

[25] Howorka K Pumprla J Jirkovska A: Modified orthostatic load for spectral analysis of short-term heart rate variability improves the sensitivity of autonomic dysfunction assessment. *J Diabetes Complications.* 2010;24(1):48–54. 10.1016/j.jdiacomp.2008.10.003 19062311

[26] Pickering TG Hall JE Appel LJ: Recommendations for blood pressure measurement in humans and experimental animals: Part 1: blood pressure measurement in humans: a statement for professionals from the Subcommittee of Professional and Public Education of the American Heart Association Council on High Blood Pressure Research. *Hypertension.* 2005;45(1):142–161. 10.1161/01.HYP.0000150859.47929.8e 15611362

[27] Body composition monitor, Instruction manual. Omron Healthcare Ltd., IM-HBF-508–E-04–05/2012. Reference Source

[28] Durnin JV Rahaman MM: The assessment of the amount of fat in the human body from measurements of skinfold thickness. *Br J Nutr.* 1967;21(3):681–9. 10.1079/BJN19670070 6052883

[29] Levy JC Matthews DR Hermans MP: Correct homeostasis model assessment (HOMA) evaluation uses the computer program. *Diabetes Care.* 1998;21(12):2191–2. 10.2337/diacare.21.12.2191 9839117

[30] Vinik A Ziegler D: Diabetic cardiovascular autonomic neuropathy. *Circulation.* 2007;115(3):387–97. 10.1161/CIRCULATIONAHA.106.634949 17242296

[31] Howorka K Pumprla J Schabmann A: Optimal parameters of short-term heart rate spectrogram for routine evaluation of diabetic cardiovascular autonomic neuropathy. *J Auton Nerv Syst.* 1998;69(2–3):164–72. 10.1016/S0165-1838(98)00015-0 9696273

[32] Havel PJ: Control of energy homeostasis and insulin action by adipocyte hormones: leptin, acylation stimulating protein, and adiponectin. *Curr Opin Lipidol.* 2002;13(1):51–9. 11790963 10.1097/00041433-200202000-00008

[33] Mayerson AB Hundal RS Dufour S: The effects of rosiglitazone on insulin sensitivity, lipolysis, and hepatic and skeletal muscle triglyceride content in patients with type 2 diabetes. *Diabetes.* 2002;51(3):797–802. 10.2337/diabetes.51.3.797 11872682 PMC2995527

[34] Canale MP Manca di Villahermmosa S Martino G: Obesity-related metabolic syndrome: mechanisms of sympathetic overactivity. *Int J Endocrinol.* 2013;2013:1–12. 10.1155/2013/865965 24288531 PMC3833340

[35] Straznicky NE Grima MT Sari CI: Neuroadrenergic dysfunction along the diabetes continuum: a comparative study in obese metabolic syndrome subjects. *Diabetes.* 2012;61(10):2506–2516. 10.2337/db12-0138 22664956 PMC3447913

[36] Greenfield JR Campbell LV: Role of the autonomic nervous system and neuropeptides in the development of obesity in humans: targets for therapy? *Curr Pharm Des.* 2008;14(18):1815–20. 10.2174/138161208784746716 18673184

[37] Frontoni S Bracaqlia D Gigli F: Relationship between autonomic dysfunction, insulin resistance and hypertension, in diabetes. *Nutr Metab Cardiovasc Dis.* 2005;15(6):441–449. 10.1016/j.numecd.2005.06.010 16314230

[38] Bray G: Obesity, a disorder of nutrient partitioning: the MONA LISA hypothesis. *J Nutr.* 1991;121(8):1146–1162. 1861165 10.1093/jn/121.8.1146

[39] Schroeder EB Liao D Chambless LE: Hypertension, blood pressure, and heart rate variability: the Atherosclerosis Risk in Communities (ARIC) study. *Hypertension.* 2003;42(6):1106–11. 10.1161/01.HYP.0000100444.71069.73 14581296

[40] Singh JP Larson MG O’Donnell CJ: Association of hyperglycemia with reduced heart rate variability (The Framingham Heart Study). *Am J Cardiol.* 2000;86(3):309–312. 10.1016/S0002-9149(00)00920-6 10922439

[41] Shaffer F McCraty R Zerr CL: A healthy heart is not a metronome: an integrative review of the heart’s anatomy and heart rate variability. *Front Psychol.* 2014;5:1–19. 10.3389/fpsyg.2014.01040 25324790 PMC4179748

[42] Moore R Groves D Nolan J: Altered short term heart rate variability with spinal cord stimulation in chronic refractory angina: evidence for the presence of procedure related cardiac sympathetic blockade. *Heart.* 2004;90(2):211–212. 10.1136/hrt.2002.002998 14729802 PMC1768050

[43] Wijers SL Saris WH van Marken Lichtenbelt WD: Recent advances in adaptive thermogenesis: potential implications for the treatment of obesity. *Obes Rev.* 2009;10(2):218–226. 10.1111/j.1467-789X.2008.00538.x 19021870

[44] Howorka K Duric D Howorka N: Proof-of-concept fuer cold-induced thermogenesis: retrospektive Datenanalyse bei Diabetes mellitus und abdominellem Uebergewicht. *Wien Klin Wochenschr.* 2014;17–18/14(14):596.

[45] Yoneshiro T Aita S Matsushita M: Brown adipose tissue, whole-body energy expenditure, and thermogenesis in healthy adult men. *Obesity (Silver Spring).* 2011;19(1):13–16. 10.1038/oby.2010.105 20448535

[46] Zafir B: Brown adipose tissue: research milestones of a potential player in human energy balance and obesity. *Horm Metab Res.* 2013;45(11):774–785. 10.1055/s-0033-1348264 23803970

[47] Scalzo RL Peltonen GL Giordano GR: Regulators of human white adipose browning: evidence for sympathetic control and sexual dimorphic responses to sprint interval training. *PLoS One.* 2014,9(3):e90696. 10.1371/journal.pone.0090696 24603718 PMC3946216

[48] Despres JP Lemieux I Bergeron J: Abdominal obesity and the metabolic syndrome: contribution to global cardiometabolic risk. *Arterioscler Thromb Vasc Biol.* 2008;28(6):1039–49. 10.1161/ATVBAHA.107.159228 18356555

[49] Cerhan JR Moore SC Jacobs EJ: A pooled analysis of waist circumference and mortality in 650,000 adults. *Mayo Clin Proc.* 2014;89(3):335–45. 10.1016/j.mayocp.2013.11.011 24582192 PMC4104704

[50] de Koning L Merchant AT Pogue J: Waist circumference and waist-to-hip ratio as predictors of cardiovascular events: meta-regression analysis of prospective studies. *Eur Heart J.* 2007;28(7):850–6. 10.1093/eurheartj/ehm026 17403720

[51] Pumprla J Howorka K Kolackova Z: Data of non-contact radiofrequency-induced reduction of abdominal fat HRV. *F1000Research.* 2014 Data Source 10.12688/f1000research.5708.1 PMC4431383 26069728

