# Supplementary material for: Non-contact radiofrequency-induced reduction of subcutaneous abdominal fat correlates with initial cardiovascular autonomic balance and fat tissue hormones: safety analysis
Source: F1000Res. 2015 Feb 20;4:49. [Version 1] doi: 10.12688/f1000research.5708.1 (PMC4431383; doi:10.12688/f1000research.5708.1)
Supplement: Peer review history

# Peer review: Referee response for version 1

**DOI:** 10.5256/f1000research.6103.r8553

**Published:** 2015-05-06

**Authors:** Andreas Thomas

**Affiliations:** 1 Diabetes Division, Medtronic GmbH, Meerbusch, Germany

This study shows the success of the radiofrequency-induced reduction of abdominal fat, while the correlation with the HRV changes (VLF spectral band). This is a very interesting study confirming the important link between obesity and cardiovascular risk parameters. It could discuss whether the HRV measurement is a good marker for the success of weight loss. It should be noted that the HRV measurement is highly selective, but not very specific. The only downside is that in the sense of a "proof of concept" study, the evidence is not high. The results should be confirmed with a randomized controlled trial.

Congratulations to the authors!

I have read this submission. I believe that I have an appropriate level of expertise to confirm that it is of an acceptable scientific standard.

# Peer review: Referee response for version 1

**DOI:** 10.5256/f1000research.6103.r7774

**Published:** 2015-03-18

**Authors:** Klaus Fritz

**Affiliations:** 1 Department of Dermatology, Bern University, Bern, Switzerland

The study shows data that were previously missing, on a therapy that increases temperature of the body and especially of the tissue treated. Data were missing on whether this might be a danger for cardiovascular diseases, circulation, or metabolism. These missing data are presented here for the first time, showing that the RF treatment described in the article is safe.

I have read this submission. I believe that I have an appropriate level of expertise to confirm that it is of an acceptable scientific standard.
